# Functional Outcome of Pediatric Subtrochanteric Fractures Treated With a Titanium Elastic Nailing System (TENS) Versus Plating

**DOI:** 10.7759/cureus.40036

**Published:** 2023-06-06

**Authors:** Nandini Sanjay, Hariprasad Seenappa, Arun H Shanthappa, Vinod Kumar K

**Affiliations:** 1 Department of Orthopaedics, Sri Devaraj Urs Academy of Higher Education and Research, Kolar, IND; 2 Department of Orthopaedics, Sri Devaraj Urs Academy Of Higher Education and Research, Kolar, IND

**Keywords:** malalignment, plating, titanium elastic nailing, subtrochanteric, paediatric femur fracture

## Abstract

Introduction

Uncertainty exists regarding the ideal course of therapy for subtrochanteric fractures in children of intermediate age. These fractures are challenging to treat, with scarce literature-based evidence to support a definitive implant. The ideal course of treatment should consider the patient's weight, age, femoral canal size, associated injuries, fracture stability, and surgeon's experience. A subtrochanteric femoral fracture in a child between the age of 5-12 is difficult to treat. For these patients, there is debate concerning the optimal internal fixation, hence this study was conducted to try and determine the superior mode of treatment for these fractures. The objective of this study is to compare functional outcomes of subtrochanteric fractures in the paediatric age group operated on with titanium elastic nail and plate fixation and the complications associated with both treatment modalities.

Materials and methods

This is a retrospective observational study of 40 cases that were admitted and operated on in the hospital of the current study from May 2007 to November 2021. Twenty patients underwent titanium elastic nailing system (TENS) nailing and the other 20 patients underwent plating for subtrochanteric fractures. The surgeries were conducted at our institute and patients were followed up at one-, three-, and six-month intervals. The final functional results were calculated with the help of the Flynn scoring system.

Results

Out of 40 patients involved in the present study, 17 were female while 23 were male. Twenty patients received treatment with titanium elastic nails, and the remaining twenty received plating. The majority of the patients were males around 9.6 years of age on average in the plating group and 8.9 years in the nailing group. In comparison to 75% of participants in the plating group, 40% of patients who received nailing showed excellent results. Results were satisfactory for five patients who received titanium elastic nails and one who received plating. The only poor outcomes were noticed in six people (30%) in TENS and three people (15%) in the plating group who went through unplanned surgery for complications. In comparison to the plating group, the overall rate of complication was much greater in the TENS group.

Conclusion

We would like to conclude our study that, in accordance with Flynn's score, both elastic nailing and plating stabilization can produce positive functional outcomes. Both groups have a similar percentage of excellent and good results. We also conclude that the overall complication rate is slightly higher for patients treated with TENS when compared to plating for subtrochanteric fractures.

## Introduction

Fractures in the subtrochanteric region of the femur are rare and account for 4-10% of paediatric femur fractures [1]. The best therapeutic strategy for these fractures in children of adolescent age is unknown. They are a special type of injury when the proximal fragment is abducted, flexed, and externally rotated as a result of the movements of the abductor group, iliopsoas, and external rotator muscles respectively. In addition, subtrochanteric femur fractures TENS are linked with intricate fracture patterns. For the above reasons, it is difficult to attain and maintain a reduction in these fractures by “non-operative” techniques.

These fractures are challenging to treat, with scarce literature-based evidence to support a definitive implant. No significant consensus exists among authors about the definition of femur fracture of subtrochanteric in children. The ideal course of treatment should consider the patient's weight, age, fracture stability, femoral canal size, associated injury, and experience of the surgeon [2]. According to previous research, using TENS to treat children with proximal 3rd of femur fractures and length-unstable fractures carries a greater risk of sequelae. Submuscular plating & open plating are other preferred options [3].

Some authors have included intramedullary interlocking (IMIL) nails for the treatment of these fractures in children aged eight years and above [4]. A subtrochanteric femoral fracture in a child from 5-12 age group is difficult to treat. For these patients, there is debate concerning optimal internal fixation [5]. We would like to state that even though TENS nailing is preferred due to less soft tissue dissection, plating can be proposed as the standard treatment as fewer complications occur with the latter.

## Materials and methods

Study design

This study is a retrospective observational analysis of 40 cases that were admitted and operated on in the Orthopedics Department, at R. L. Jalappa Hospital, Kolar, Karnataka, India from May 2007 to November 2021 (14 years); 20 patients underwent TENS nailing and another 20 patients underwent plating for subtrochanteric fractures. The study was approved by the Institutional Ethics Committee of Sri Devaraj Urs Medical College (DMC/KLR/IEC/359/2021-22), and all patients who were contacted for follow-up provided informed consent. Follow-up was done at one, three, and six months. Every surgery was done at our institute.

Criteria and evaluation

The inclusion criteria for this study included individuals with closed subtrochanteric fractures and an age range of 6-14 years. Patients with, treatment delay of more than three weeks, pathological fractures, neuromuscular disorders, and polytrauma patients were excluded.

The Flynn scoring system [6] was utilized to determine final functional results, and the two groups were categorized as excellent, satisfactory, or poor depending on the degree of residual leg length discrepancy, the malignment of the fracture, pain, complications, and unanticipated surgery to treat complications. The radiographic evaluation includes preoperative and postoperative anteroposterior (AP) views of the pelvis as well as lateral and AP views of the femur.

Statistical methods

Data obtained were entered using MS Excel (Microsoft, Redmond, WA, United States) and analyzed by the SPSS Standard v. 20 (IBM, Armonk, NY, United States). Depending on the distribution's normality, the median (interquartile range) or mean (standard deviation) was applied to summarise all continuous variables. Categorical variables were compiled with proportions. The chi-square test was utilized to analyze categorical outcomes (age group, type of fixation, gender, site, mechanism of injury, comorbidities, infection, outcomes (e.g., non-union, malunion, and delayed union)) between study groups. Statistics were considered significant at p-value <0.05.

## Results

A total of 40 patients met the inclusion criteria for the current study. These patients were available for follow-up and were followed up at one-, three-, six- and 12-month intervals. Two patients were available for follow-up for only six months, after which they were noncompliant for follow-up.

Demographic details of participants by their age, the affected limb, and the mode of injury are shown in Table 1 which shows that both groups were demographically similar.

**Table 1:** Demographic data SD: standard deviation, RTA: road traffic accident

	Plating Group	Titanium Elastic Nails Group	Total (N=40)	p-value
Mean (SD) age (in years)	9.6 (2.1)	8.9 (2.0)	9.2 (2.1)	0.291
Sex (number of patients)				
Male	13 (65%)	10 (50%)	23 (57.5%)	0.337
Female	7 (35%)	10 (50%)	17 (42.5%)	
Leg Affected				
Right	13 (65%)	12 (60%)	25 (62.5%)	0.744
Left	7 (35%)	8 (40%)	15 (37.5%)	
Mode of Injury				
Self-fall	17 (85%)	13 (65%)	30 (75%)	0.316
RTA	3 (15%)	7 (35%)	10 (25%)	

**Figure 1:**
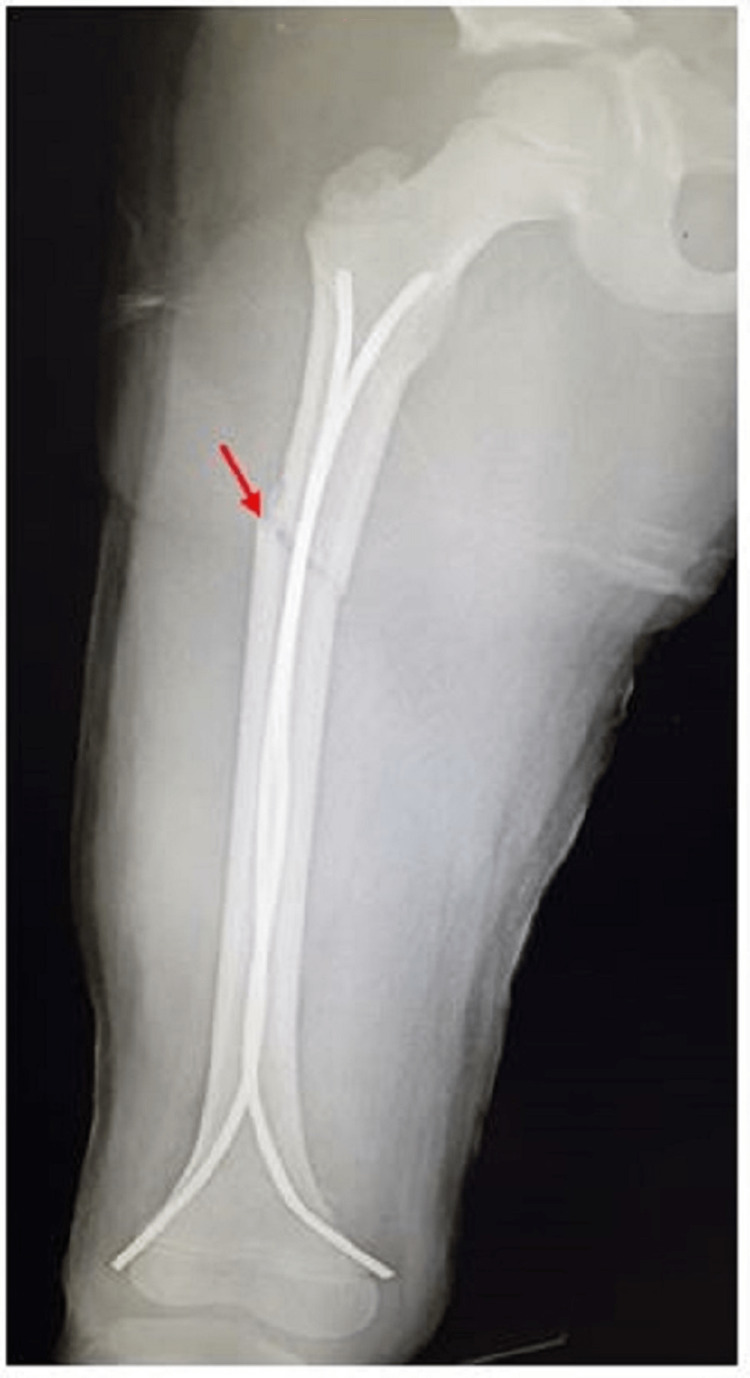
Antero-posterior radiograph of a nine-year-old male with right subtrochanteric fracture (red arrow) who underwent TENS fixation TENS: titanium elastic nailing system

**Figure 2:**
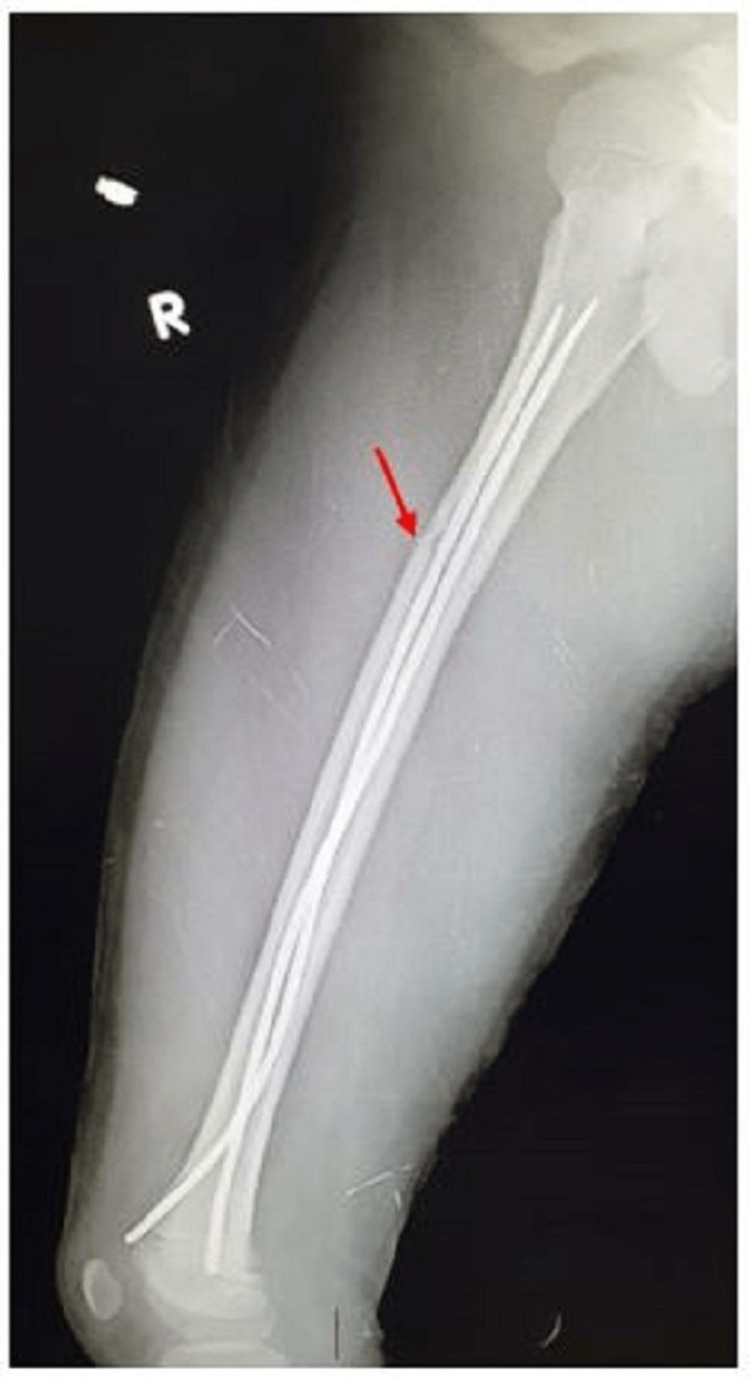
Lateral radiograph of a nine-year-old male with right subtrochanteric fracture (red arrow) who underwent TENS fixation TENS: titanium elastic nailing system

Out of 40 patients, 23 were male and 17 were female; 20 patients received treatment with nailing (Figures 1-2), and the remaining 20 received plating. The majority of patients were males of 9.6 and 8.9 years of age on average in the plating group and nailing group respectively. In regard to age, gender, and involved limb, there were no discernible differences between the two groups. There were no open fractures. The mode of injury in the majority (75%) of the study participants was self-fall while the remaining participants (25%) sustained a fracture following a road traffic accident (RTA).

When compared to patients who received plating, patients with titanium elastic nails progressed to full weight bearing relatively earlier. No significant variations were observed between the two groups in relation to the average length of hospitalization.

Follow-up results after one month are shown in Table 2, where it was observed that 10 (50%) patients in the plating group had < 1 cm shortening when compared to eight (30%) patients in the nailing group. Shortening of >2cm was noted in two patients in the nailing group. A malalignment of >10° was observed in nine patients at the end of one month (seven of which belonged to the nailing group). It was also observed that 26 (65%) patients had pain after one month of surgery, the majority of whom belonged to the nailing group.

Follow-up results after three months are shown in Table 2, where three patients in the plating group had < 1 cm shortening when compared to four (30%) patients in the nailing group. Shortening of <2 cm was noted in two patients in the nailing group but no patient had > 2 cm after three months' follow-up in either of the two groups. A malalignment of >10° was observed in three patients at the end of three months (one patient belonged to the plating group). It was observed that there was a significant reduction in pain after three months.

**Table 2:** Comparison of outcome scores between the two study groups at one and three months follow-up

	ONE MONTH FOLLOW UP				THREE MONTH FOLLOW UP			
	Plating Group	Titanium Elastic Nails Group	Total (N=40)	p-value	Plating Group	Titanium Elastic Nails Group	Total (N=40)	p-value
Leg Length Discrepancy								
No discrepancy	8 (40%)	6 (30%)	14 (35%)		17 (85%)	14 (70%)	31(77.5%)	0.296
≤ 1 cm	10 (50%)	8 (30%)	18 (45%)		3 (15%)	4 (20%)	7 (17.5%)	
≤ 2 cm	2 (10%)	4 (20%)	6 (15%)		0	2 (10%)	2 (5%)	
> 2 cm	0	2 (10%)	2 (5%)		-	-	-	
Malalignment								
≤ 5	8 (40%)	4 (20%)	12 (30%)	0.125	17 (85%)	11 (55%)	28 (70%)	0.111
6 – 10	10 (50%)	9 (45%)	19 (47.5%)		2 (10%)	7 (35%)	9 (22.5%)	
> 10	2 (10%)	7 (35%)	9 (22.5%)		1 (5%)	2 (10%)	3 (7.5%)	
Pain								
Present	11 (55%)	15 (75%)	26 (65%)	0.185	3 (15%)	6 (30%)	9 (22.5%)	0.256
Absent	9 (45%)	5 (25%)	14 (35%)		17 (85%)	14 (70%)	31 (70%)	

Outcome scores were calculated at the end of one month and were observed to be considerably greater in the plating group (Table 3). Two patients (one of each group) were lost to follow-up after six months, hence they have been excluded from the final score.

In comparison to the plating group (15%; three of 20), the total rate of complication was considerably greater in the TENS group (30%; six of 20) (Table 3). The six patients who got nailing all experienced more than one complication. In the TENS group, the most prevalent sequelae were fracture malalignment at the time of radiographic union (four patients), residual leg-length discrepancies (two patients), and pain from prominent nails (six patients). One malaligned fracture and two unequal leg lengths were among the complications in the plating group.

**Table 3:** Flynn scoring system comparing outcomes for both groups TENS: titanium elastic nailing system

Flynn Scoring	Plating group	TENS group	Total	p-value
Excellent result	15 (75%)	8 (40%)	23 (58%)	0.027
Satisfactory result	1 (5%)	5 (25%)	6(15%)	
Poor result	3 (15%)	6 (30%)	9 (22.5%)	

**Figure 3:**
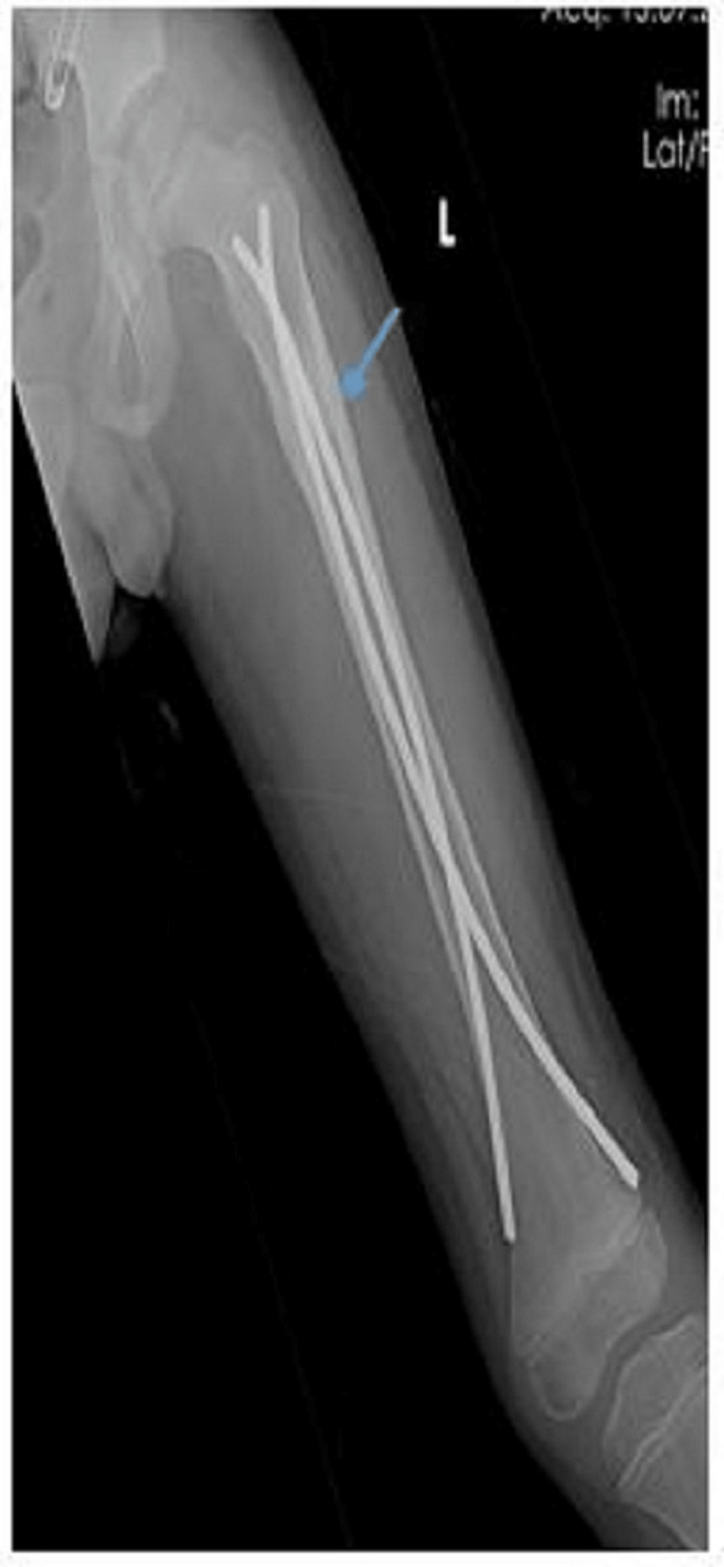
Antero-posterior radiograph of a nine-year-old male who underwent TENS fixation for subtrochanteric fracture (blue arrow) with fracture union TENS: titanium elastic nailing system

**Figure 4:**
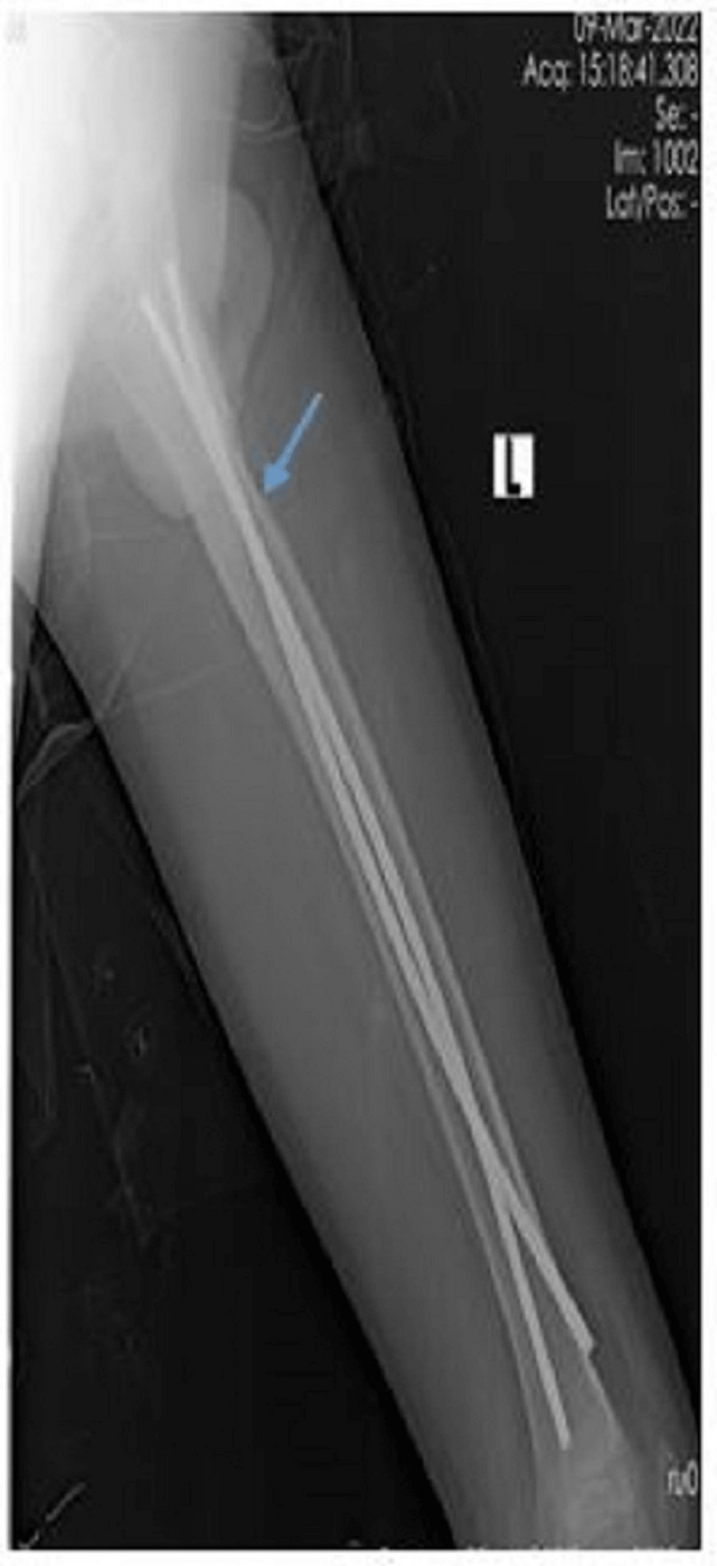
Lateral radiograph of a nine-year-old male who underwent TENS fixation for subtrochanteric fracture (blue arrow) with fracture union TENS: titanium elastic nailing system

**Figure 5:**
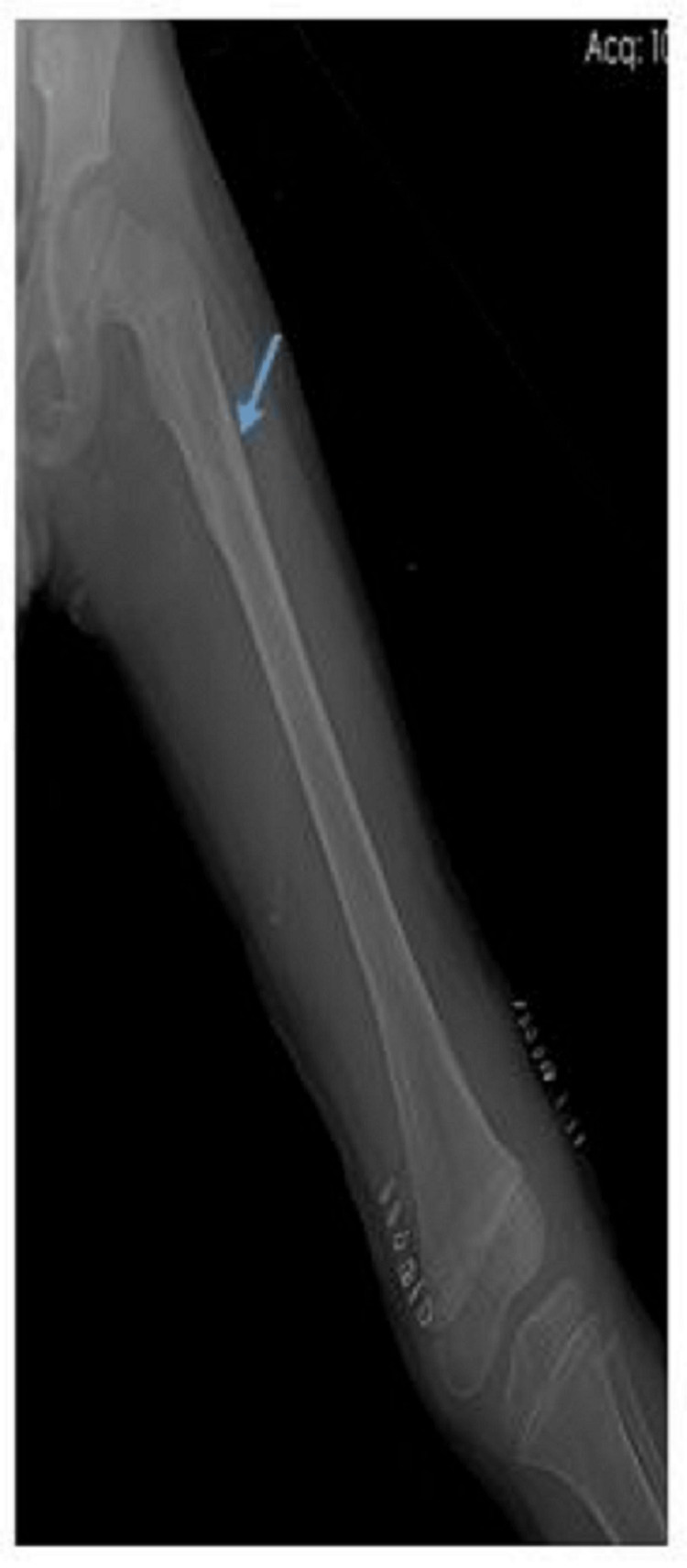
Antero-posterior radiograph of a 9-year-old male who underwent implant removal of TENS after fracture union (blue arrow)

**Figure 6:**
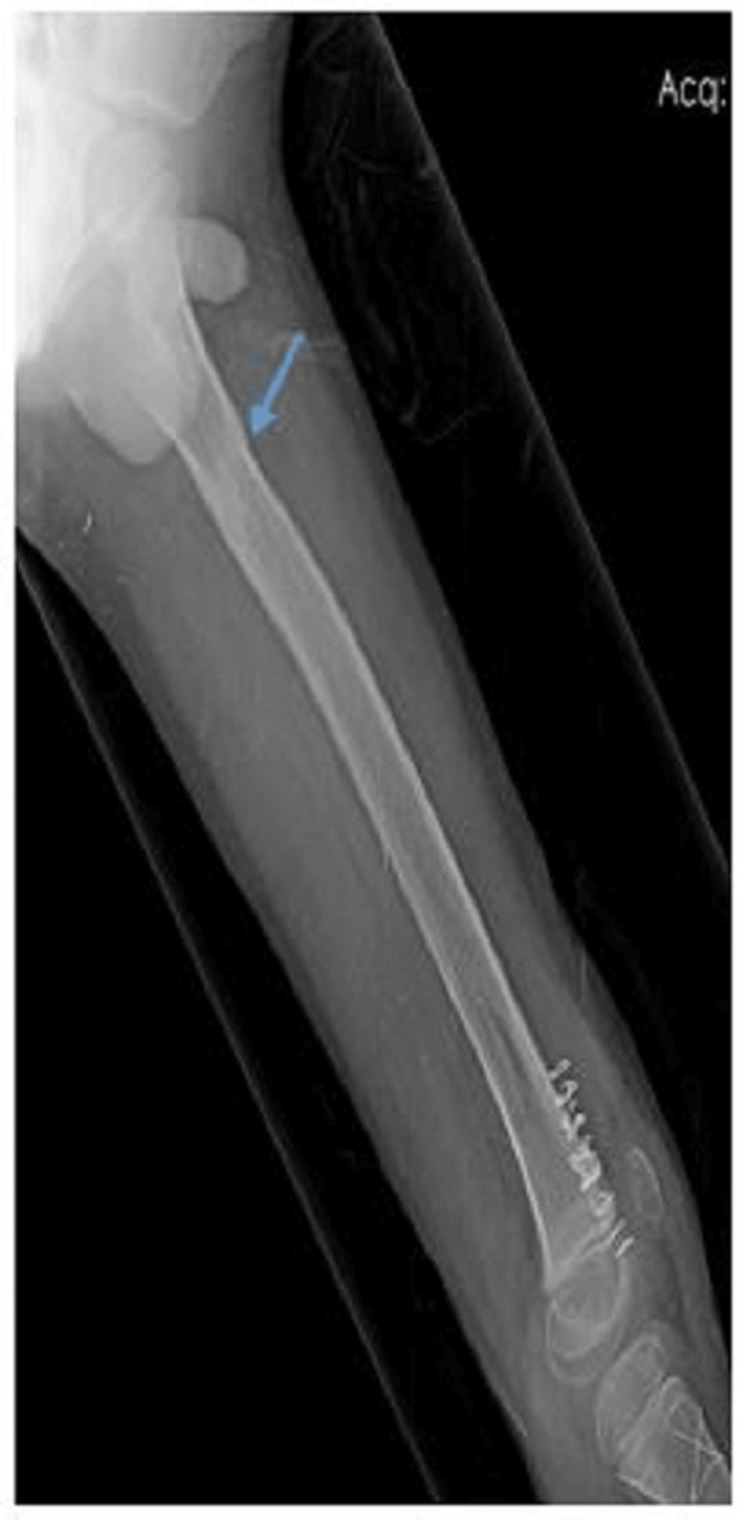
Lateral radiograph of a nine-year-old male who underwent implant removal of TENS after fracture union (blue arrow) TENS: titanium elastic nailing system

When compared to 75% of patients in the plating group, 40% of patients who received titanium elastic nails showed excellent results. The below-given figures show a follow-up patient treated with TENS. (Figures 3-6). Results were satisfactory for five patients who received TENS and only for one patient who received plating. Only six patients (30%) in the nailing group and three patients (15%) in the plating group who underwent unanticipated surgery for complications had poor outcomes. 

## Discussion

Pediatric subtrochanteric fractures are a challenge to treat. There are few studies in orthopaedic literature devoted to paediatric subtrochanteric fracture treatment, and the mainstay of care is not universally agreed upon [7]. The literature on the treatment of these fractures is either limited or divided. A lack of unanimity regarding the definition of subtrochanteric femur fractures in children is one of the main issues. A paediatric subtrochanteric femur fracture, according to Pombo and Shilt, is within 10% of the entire femur length below the lesser trochanter [8].

An alternative definition of subtrochanteric fractures was proposed by Ireland and Fisher, who said that the fracture line must be within the proximal 1/4th of the space between the adductor tubercle and the intertrochanteric region. Additionally, it was acknowledged in the literature that managing femur fractures in the subtrochanteric area presents special challenges [7]. The inability to fully correct for malalignment in the subtrochanteric area and powerful deforming muscle forces that drive the proximal fragment into abducted, flexed, and externally rotated positions make it challenging to maintain fracture reduction [9]. According to earlier research on paediatric femoral shaft fractures, satisfactory outcomes can be achieved nonoperatively with manipulation or traction and cast immobilization [7]. This is due to the high remodelling in the paediatric age group which allows acceptable angulation and shortening correction. In spite of obtaining excellent results with the above method, complications such as prolonged immobilization, knee stiffness, pin tract infection, and refracture rates have led to various other management options in these patients.

According to Jarvis et al.'s study, a subtrochanteric fracture in a skeletally immature adolescent resulted in poor clinical and radiologic results, comprising fracture malalignment with greater than 16 degrees of angulation and lower limb length shortening by 2.6 cm on average [10]. For young adolescents and school-age children with femoral shaft fractures, TENS is now the most widely used treatment [6, 11]. According to Ellis et al. [12], the distal femoral interlocking intramedullary nail offers an effective treatment for older children who have length-instable fractures since it can successfully maintain fracture reduction and prevents the development of shortening.

Recent research by Parikh et al. [13] showed that the results of 33 school-aged children having subtrochanteric fractures who received TENS treatment were satisfactory. They concluded by saying that TENS is a secure and effective choice to treat children having subtrochanteric femur fractures. The same is comparable with our study, where we noted the majority of the patients treated with TENS had excellent and satisfactory results (40% and 25% respectively). As treatment with TENS is frequently a closed reduction method, it is considered a safer method than plating as periosteal stripping is avoided and soft tissue around the fracture is minimally damaged.

Following TENS surgery, Flynn et al. found that a significant portion of proximal 3rd femur fractures had an angulation higher than 5 degrees. Similar to the result obtained in the above study, it was observed that four patients treated with TENS were noted to have malalignment > 5 degrees after the one-year follow-up [6]. Similar to the findings of earlier research [3, 5], plating received considerably higher outcome scores in the current study, but both groups revealed a significant score of excellent as well as good outcomes.

Ho et al. [14] observed a 22% rate of complications for proximal 3rd femur fractures treated with TENS. With length-unstable femur fractures treated using TENS, Narayanan et al. [15] and Sink et al. [16] demonstrated a rise in the rate of complications and risk of unanticipated revision surgery.

In light of the aforementioned findings, additional internal fixation techniques should soon replace TENS to manage paediatric subtrochanteric femoral fractures.

James et al. [17], in a research conducted in South India, comparing TENS and plating in paediatric diaphyseal fractures, stated that plating and TENS are equally viable, safe, and effective ways to treat paediatric diaphyseal femoral fractures.

According to Li et al. [3], plating was a better treatment option than TENS fixation for subtrochanteric fractures in school-age children. Plate fixation was demonstrated as a viable option in treating paediatric femoral shaft fractures [18].

According to Sink et al., 27 children having length-unstable femur fractures treated by submuscular plating had excellent outcomes [19]. When treating unstable fractures with submuscular plating as opposed to titanium elastic nails, Sink et al. [20] also found a considerable reduction in overall and serious complications.

Similar results were obtained in the present study where excellent outcome was obtained in 75% of patients treated with plating.

A 4% complication incidence was discovered by Kanlic and colleagues [21] in 51 paediatric submuscular plating-treated femoral shaft fractures. In 60 paediatric patients with compression-plate-treated femoral shaft fractures, Caird and colleagues [22] found a 10% rate of complications. Comparable results were obtained in our study where the complication rate of plating of subtrochanteric fractures in the study age group was 15%.

Limitations

Our study has certain limitations. The first drawback is the tiny patient population in each category. These numbers were probably insufficient to show any meaningful differences between the two groups. Our use of the Flynn scoring system to categorize the results of subtrochanteric femur fractures treated by plating is our second restriction. Although it permitted us to evaluate the results of the two groups directly, this score was not intended to evaluate the effects of plating and was not proven effective across research or treatment modalities. The fact that this study is retrospective is another drawback.

## Conclusions

We would like to conclude our study that, in accordance with Flynn's score, both elastic nailing and plating stabilization can produce positive functional outcomes. Both groups have a similar percentage of excellent and good results. Additionally, we also conclude that, when compared to plating for subtrochanteric fractures, the overall complication rate is marginally higher for patients receiving TENS (fracture malalignment at the time of radiographic union, residual leg-length inequality, and pain from prominent nails).
